# Advances in Processing Agri-Food and Aquatic By-Products and Residues for New Food Product Development

**DOI:** 10.3390/foods15142556

**Published:** 2026-07-20

**Authors:** Alcina M. M. B. Morais, Rui M. S. C. Morais

**Affiliations:** CBQF—Centro de Biotecnologia e Química Fina—Laboratório Associado, Escola Superior de Biotecnologia, Universidade Católica Portuguesa, Rua Diogo Botelho 1327, 4169-005 Porto, Portugal

## 1. Overview: The Rise of By-Product Valorization in Food Science

Food systems frequently generate huge quantities of by-products and residues at each stage of the production chain, from agricultural harvesting and aquatic processing to fermentation and industrial refining. According to the United Nations Environment Programme (UNEP) Food Waste Index Report 2024, approximately 1.05 billion tons of food were wasted in 2022 at the retail, food service, and household levels alone, while an additional 13% of global food production was lost between harvest and retail [1]. Food loss and waste collectively account for an estimated up to 10% of annual global greenhouse gas emissions, nearly five times the total emissions from the aviation sector, and occupy around 28% of the world’s agricultural land [1,2]. Historically considered waste streams, these impose substantial environmental and economic burdens, contributing to greenhouse gas emissions, nutrient loss, and disposal costs. Yet, they are simultaneously rich in proteins, lipids, polysaccharides, pigments, and bioactive compounds of considerable nutritional and potential functional value.

In the past decade, the convergence of circular bioeconomy principles, advances in green extraction technologies, and increasing consumer demand for sustainable and functional foods has promoted a paradigm shift: by-products are becoming increasingly viewed not as liabilities but as underexploited resources [3,4]. Fish skin and bones, spent brewer’s yeast, seaweed and microalgal biomasses and residues, plant bagasses, and plant fermentation liquids have each attracted significant research interest as feedstocks for novel food ingredients and packaging materials [5]. Parallel developments in food technologies, e.g., ultrasound (US)-assisted extraction and dehydration, pulsed electric fields (PEF), fermentation engineering, and encapsulation, have expanded the tools available for transforming these residues into market-ready products [3,6,7].

Despite these considerations, the field remains characterized by heterogeneity in methodological approaches, a lack of or inconsistent scaling pathways, and fragmented knowledge across commodity sectors. Transitioning from proof of concept to commercial application is hindered by gaps in bioactivity characterization, sensory optimization, regulatory alignment, and life-cycle sustainability assessment [3,4]. This Special Issue (SI) was conceived to address these challenges precisely by assembling contributions that advance both the science and the practical applicability of agri-food and aquatic by-product valorization.

## 2. Gaps in Knowledge and How This Special Issue Addresses Them

Despite the growing literature on by-product valorization, several critical gaps have limited its advancement. First, the functional characterization of extracted biopolymers, particularly regarding mechanical, barrier, and bioactive properties under a food-relevant perspective, has remained incomplete for many underutilized species. Contribution 1 addresses this directly by extracting and characterizing gelatin from Tambatinga (*Colossoma macropomum* × *Piaractus brachypomus*) fish skin. The films prepared with this gelatin demonstrate high UV-blocking capacity, favorable tensile strength, and reduced water vapor permeability, thereby establishing a strong potential for sustainable packaging applications. This work aligns with broader research interest in fish-derived gelatin as a biodegradable and circular biopolymer [8].

The scalability of bioactive fraction recovery from microbial and yeast residues has also not been adequately demonstrated beyond laboratory settings. Contribution 2 filled this gap by developing a reproducible pilot-scale fractionation protocol for peptide-rich extracts from brewer’s spent yeast (BSY), confirming significant antioxidant activity (validated by ABTS and ORAC assays) and successful incorporation into protein-enriched crackers. The retentate from the 10 kDa ultrafiltration fraction at pilot scale exhibited superior bioactivity, providing industry-relevant validation absent in the prior BSY literature [9].

In addition, sensory acceptance of algae- and microalgae-derived ingredients in food formulations remains a well-acknowledged barrier. Contribution 3 responds to this challenge through lactic acid fermentation with *Lactiplantibacillus plantarum*, demonstrating that controlled bioprocessing can improve the volatile and flavor profiles of microalgal biomass. Notably, it is possible to reduce aldehyde compounds such as hexanal, which is responsible for fishy odors [10]. Clean-label vegan burgers produced from fermented microalgal biomasses presented an acceptable color and texture, providing a replicable formulation strategy for algal ingredient incorporation. In a complementary approach, contribution 4 leveraged *Chlorella vulgaris* lipid extracts, obtained through non-thermal high-pressure extraction (HPE), to partially substitute fat in two bakery products (brioche and rice cake). This met nutritional targets without compromising consumer acceptability, an area where prior evidence had been mostly anecdotal. The importance of sensorial acceptability is also brought up by contribution 5, which proposed reusing processing waste from the Sicilian almond (*Prunus amygdalus* Batsch.) cultivar Tuono to formulate a new functional baked product (muffin) that is gluten- and lactose-free. Muffins enriched with 6% almond skin showed the highest total phenols (TPC) and flavonoids (TFC), exerting a promising antioxidant activity as an inhibitor of lipid peroxidation and a promising α-glucosidase inhibitory effect. These results, supported by sensory analysis, show that muffins enriched with almond skin have potential as a new functional bakery product.

Another important issue is that the mechanistic understanding of fermentation-induced bioactive changes in discarded liquid effluents has been insufficient to support process design. Contribution 6 addresses this by analyzing metabolic changes in phenolic and peptide profiles during *Pediococcus pentosaceus* fermentation of bamboo shoot boiling liquid (BSBL), a processing effluent ordinarily discarded as wastewater, but which is very rich in organic load. Using partial least squares discriminant analysis (PLS-DA), the authors elucidate how bioactive enrichment and bitterness reduction co-occur within the first 24 h of fermentation, identifying phenylpropanoid metabolites, such as cinnamic acid and 5,7-dimethoxyflavone, as key compounds. This constitutes critical knowledge for the development of functional fermented beverages from normatively discarded processing effluents.

The processing efficiency of high-moisture by-products, which is a restrictive point in economic feasibility, has received limited rigorous treatment. Contribution 7 quantifies the impact of US and PEF pretreatments on convective drying of cassava bagasse, achieving reductions in drying time of up to 72% with probe ultrasound at 26 kHz relative to conventional convective drying alone (3.83 h vs. 14.0 h). PEF at 7.5 kV/cm also achieved a 52.4% reduction in drying time. This contribution directly addresses logistical and energy-cost barriers that have prevented industrial starchy by-product valorization.

Finally, integrated valorization strategies that simultaneously generate food ingredients and agricultural inputs have been underexplored. Contribution 8 demonstrates an elegant dual-pathway approach using *Fucus spiralis* seaweed: polysaccharides and bioactives are extracted via microwave hydrodiffusion and gravity (MHG) for edible film production, yielding films with significant antioxidant ABTS and DPPH values, while residual biomass is valorized as a biostimulant promoting seed germination and seedling development. Biostimulants with polysaccharides enhanced pea germination by 48%, while those without polysaccharides increased rice and tomato root dry weight by 53% and up to 176%, respectively. This represents a meaningful contribution to the integration of food and agri-systems valorization, and deserves considerably more attention.

Overall, the contributions to this SI span a diversity of matrices—animal, plant, microbial, and algal—and collectively demonstrate that targeted, evidence-based research can systematically close the translational gaps between by-product biomass and functional food product development.

## 3. Future Research Priorities

While the contributions in this SI advance the field substantially, they also help to identify areas for future research. The following priorities are identified as most consequential for accelerating the responsible commercial release of agri-food and aquatic by-product valorization.

### 3.1. Scaling and Process Engineering

The majority of valorization studies, including several in this SI, remain at the laboratory or pilot scale. Rigorous process simulations and techno-economic analyses are urgently needed to evaluate the feasibility of scale-up under realistic production setups [3]. Future work should address yield variability across feedstock batches, energy demands of emerging technologies such as PEF and US, and integration into existing industrial processing lines. In particular, continuous-flow extraction of bioactive compounds and real-time quality monitoring systems represent promising engineering directions.

### 3.2. Bioavailability and In Vivo Functional Validation

Demonstrating antioxidant activity, antimicrobial potential, or nutritional enrichment in vitro is a necessary but insufficient foundation for health claims. Future research must prioritize in vivo bioavailability studies and clinical or pre-clinical validation of the functional effects attributed to by-product-derived bioactives [4]. This is especially critical for peptide fractions, polysaccharides, and pigment compounds whose bioaccessibility can be profoundly altered during gut digestion or food matrix interaction. Without such evidence, regulatory pathways for novel food ingredient approval will not be possible. Technologies such as micro- and nanoencapsulation serve to protect bioactive compounds and to control the release, and assure their delivery through the gastrointestinal tract [7].

### 3.3. Life-Cycle Assessment and Environmental Sustainability

The environmental credentials of valorization processes must be empirically established rather than assumed. Comprehensive life-cycle assessments (LCA) covering energy consumption, water use, compound toxicity, and transport logistics are necessary to verify if valorization really reduces the environmental burden relative to conventional waste disposal [5]. Of particular concern are energy-intensive drying processes and solvent-based extractions, which may overcome the sustainability gains of residue diversion. Comparative LCA across competing processing routes will be essential for evidence-based process selection.

### 3.4. Regulatory Frameworks and Novel Food Classification

The regulatory landscape governing novel food ingredients derived from by-products and residues, particularly those from non-traditional species, fermentation of waste streams, or algal biomass, varies considerably across jurisdictions and remains a significant barrier to market entry [11]. Under Regulation (EU) 2015/2283 on novel foods, foods and food ingredients that were not consumed to a significant degree within the European Union before 15 May 1997 are subject to a centralized authorization procedure [12]. Future research should engage proactively with regulatory issues, developing safety dossiers and data that support novel food applications. The EU Novel Food Catalogue, updated in February 2024, provides growing guidance on the regulatory status of seaweed and microalgae species [12]. Collaborative platforms between academia, industry, and regulatory agencies will be vital in this regard.

### 3.5. Consumer Acceptance and Market Positioning

Sensory acceptability and consumer perception remain underinvestigated relative to compositional and functional characterization. Future studies should incorporate structured consumer trials, Willingness-to-Pay (WTP) analyses, and co-design methodologies to understand the conditions under which by-product/residue-derived ingredients gain market acceptance. Particular attention should be paid to transparency in labeling, the communication of sustainability credentials, and segment-specific positioning (e.g., sports nutrition, plant-based, functional food).

### 3.6. Integrated Circular Valorization Platforms

The most impactful future direction may be the design of integrated biorefinery models, in which several products are recovered from a single feedstock in a complementary manner [3] (contribution 7). The *Fucus spiralis* example in this Issue, extracting bioactives for edible films while repurposing residual biomass as a biostimulant, illustrates this potential (contribution 8). Extending such integrated approaches to animal processing residues, cereal by-products, and fermentation effluents could substantially improve the economic viability of valorization solutions while minimizing residual waste. Linking food, packaging, feed, and agricultural applications within unified circular models should be a central aspiration for the next generation of by-product research [4].

### 3.7. Digital Tools, Artificial Intelligence, and Predictive Modeling

Emerging computational approaches, including machine learning models for bioactive prediction, digital twins for process optimization, and AI-assisted formulation design, offer transformative potential for by-product and residue valorization research [3]. These tools can accelerate screening of feedstock diversity, optimize extraction conditions, and predict sensory outcomes, thereby dramatically reducing the experimental load of experimental optimization. Future research programs should actively encourage the integration of data science methodologies into valorization workflows.

## 4. Conclusions

This SI on Advances in Processing Agri-Food and Aquatic By-Products and Residues for New Food Product Development represents a timely and substantive contribution to a field of growing scientific, industrial, and societal importance. The assembled research demonstrates that the valorization of underutilized biomass streams is not only conceptually sound but technically achievable across diverse matrices and processing platforms. Collectively, these contributions advance our understanding of the functional properties of recovered ingredients, the processes that transform by-products and residues into food-grade components, and the formulation strategies that enable their successful integration into consumer products.

We hope that the knowledge assembled here will catalyze further interdisciplinary collaboration and accelerate the transition toward more sustainable, circular agri-food systems.

## Data Availability

No new data were created or analyzed in this study. Data sharing is not applicable to this article.
